# Phosphoproteomic profiling of early rheumatoid arthritis synovium reveals active signalling pathways and differentiates inflammatory pathotypes

**DOI:** 10.1186/s13075-024-03351-4

**Published:** 2024-06-12

**Authors:** Cankut Çubuk, Rachel Lau, Pedro Cutillas, Vinothini Rajeeve, Christopher R. John, Anna E. A. Surace, Rebecca Hands, Liliane Fossati-Jimack, Myles J. Lewis, Costantino Pitzalis

**Affiliations:** 1 Centre for Experimental Medicine and Rheumatology, William Harvey Research Institute, Queen Mary University of London and Barts NIHR BRC & NHS Trust, Charterhouse Square, London, EC1M 6BQ UK; 2 Cell Signalling and Proteomics Group, Centre for Genomics and Computational Biology, Barts Cancer Institute, Queen Mary University of London, London, EC1M 6BQ UK; 3 IRCCS Istituto Clinico Humanitas, Via Manzoni 56, Rozzao, Milan Italy

## Abstract

**Background:**

Kinases are intracellular signalling mediators and key to sustaining the inflammatory process in rheumatoid arthritis (RA). Oral inhibitors of Janus Kinase family (JAKs) are widely used in RA, while inhibitors of other kinase families e.g. phosphoinositide 3-kinase (PI3K) are under development. Most current biomarker platforms quantify mRNA/protein levels, but give no direct information on whether proteins are active/inactive. Phosphoproteome analysis has the potential to measure specific enzyme activation status at tissue level.

**Methods:**

We validated the feasibility of phosphoproteome and total proteome analysis on 8 pre-treatment synovial biopsies from treatment-naive RA patients using label-free mass spectrometry, to identify active cell signalling pathways in synovial tissue which might explain failure to respond to RA therapeutics.

**Results:**

Differential expression analysis and functional enrichment revealed clear separation of phosphoproteome and proteome profiles between lymphoid and myeloid RA pathotypes. Abundance of specific phosphosites was associated with the degree of inflammatory state. The lymphoid pathotype was enriched with lymphoproliferative signalling phosphosites, including Mammalian Target Of Rapamycin (MTOR) signalling, whereas the myeloid pathotype was associated with Mitogen-Activated Protein Kinase (MAPK) and CDK mediated signalling. This analysis also highlighted novel kinases not previously linked to RA, such as Protein Kinase, DNA-Activated, Catalytic Subunit (PRKDC) in the myeloid pathotype. Several phosphosites correlated with clinical features, such as Disease-Activity-Score (DAS)-28, suggesting that phosphosite analysis has potential for identifying novel biomarkers at tissue-level of disease severity and prognosis.

**Conclusions:**

Specific phosphoproteome/proteome signatures delineate RA pathotypes and may have clinical utility for stratifying patients for personalised medicine in RA.

**Supplementary Information:**

The online version contains supplementary material available at 10.1186/s13075-024-03351-4.

## Background

Rheumatoid arthritis (RA) is an incurable chronic inflammatory disease that leads to disability, systemic complications, early death, and places a substantial socioeconomic burden on society [1]. Although considerable progress has been made by a combination of early disease intervention and targeted biologic therapies, long term remission is elusive and response to therapy is heterogenous and difficult to accurately predict.

Most biomarker methods measure mRNA or protein levels in tissue or blood, but often give little or no direct information about whether proteins are active or inactive. Phosphoproteomic analysis has the potential to quantify whether enzymes and adaptors which regulate cell signalling pathways are in an active or inactive state. Thus, high levels of activity in specific signalling pathways in disease tissue may well relate to disease severity and outcome as well as responsiveness to therapeutics which target those active signalling pathways. One of the first clinically useful applications of phosphoproteomics has been in oncology [2], where mass spectrometry-based phosphoproteomic analysis of blast cells from acute myeloid leukaemia patients was used to dissect mechanisms of drug resistance to kinase inhibitors [3, 4].

Kinases are key signalling mediators and are known to be important in driving production of pro-inflammatory cytokines and continuation of pathobiological processes in RA [5, 6]. Some of the primary kinases involved in RA pathogenesis are candidate or existing drug targets, which include Janus Kinases (JAKs), Mitogen Activated Protein Kinases (MAPKs), Phosphoinositide 3-Kinases (PI3Ks) and Cyclin Dependent Kinases (CDKs). The JAK-Signal Transducer And Activator Of Transcription (STAT) signalling pathway is critical to cytokine signalling downstream of multiple cytokine receptors including the interferon receptors, interleukin-6 (IL-6) receptor and many others which play important roles in RA pathogenesis [7]. In 2012, tofacitinib, a small molecule JAK inhibitor, was the first kinase inhibitor approved for RA treatment [8].

The MAPKs consist of 3 primary families, the c-Jun N-terminal kinases (JNK), p38 kinases, and extracellular signal-regulated kinases (ERK) [5]. These kinases are activated by pro-inflammatory cytokines and are involved in regulation of cell proliferation, dynamic balance of pro- and anti-inflammatory signals, and matrix regulation by metalloproteinases that are the main contributors of pathologies, such as arthritis and cancer. PI3Ks generate lipid-based second messengers that control intracellular signalling pathways and have important immunological roles in mast cells, neutrophils, dendritic cells, B cells, and T cells [9]. Recent work has demonstrated targeting PI3K isoforms can suppress the progression of RA in mouse models [10]. Cell cycle regulation is another potential target for RA since a characteristic of RA is overgrowth of synovial fibroblasts, which has led researchers to explore targeting the CDKs and CDK4/6 inhibitors have been shown to suppress synovial hyperplasia in animal models [11], while the results of a phase 1b/2a trial aimed at establishing safety and maximal tolerated dose of Seliciclib, an orally available CDK inhibitor in patients with active rheumatoid arthritis, has been reported [12].

With the ongoing development of several kinases as drug targets in RA and the heterogeneity in response to therapy, we sought to examine whether different RA pathotypes showed distinct proteomic and phosphoproteomic profiling signatures. Our aim was to determine whether proteomic and/or phosphoproteomic analysis would be of utility for personalised targeted therapy strategies. It has previously been demonstrated from global gene expression analysis that the RA synovium classifies into lympho-myeloid (lymphoid), diffuse myeloid and pauci-immune fibroid pathotypes [13]. The lymphoid subtype is the most immunologically active, with enrichment in T and B cell receptors, antigen processing and presentation, and lymphocyte activation pathways. The myeloid pathotype, was more enriched in innate immune pathways, with Toll-like and NOD-like receptor signalling, and Fc gamma mediated phagocytosis pathways upregulated [14]. The fibroid pathotype was enriched with TGF-β and SMAD binding pathways [15]. Notably these pathways associated with prognosis and initial response to treatment [13, 16]. Recent single cell studies suggest that even more subgroups of RA can be defined at the synovial cellular level [17].

In this pilot study, we used protein samples extracted from treatment naïve synovial biopsies derived from the Pathobiology of Early Arthritis cohort (PEAC) study [13] to conduct phosphoproteomic and proteome profiling. We determined differential signalling and protein expression signatures between the lymphoid and myeloid RA pathotypes and correlated these against clinical features.

## Methods

### Patient samples, clinical assessment and synovial histology

Histopathology based pathotype annotation was used in the sample selection. The demographics of the samples and according to pathotype are detailed in Supplementary Table 1. RA synovial biopsies were obtained from patients as part of the Medical Research Council (MRC) funded multi-centre Pathobiology of Early Arthritis Cohort (PEAC) as described in Lewis *et al*^*13*^. The study received ethical approval from the UK Health Research Authority (REC 05/Q0703/198, National Research Ethics Service Committee London – Dulwich). All patients gave written informed consent. Clinical assessments and synovial histology were undertaken as described in the aforementioned paper.

### Optimisation

Although ultrasound-guided needle biopsy of synovium is a reliable technique to obtain high-quality synovial tissue, tissue preservation conditions and quantity of the tissue are important factors that have an impact on protein sample preparation for mass spectrometry experiments. In this pilot study, we also performed an optimisation experiment to define the optimal tissue-specific preservation solution, extraction method and minimum amount of the protein extracts required to ensure adequate protein and phosphosite identifications. We investigated the sample quality using different tissue preservation methods (snap frozen and RNALater), protein extraction methods (AllPrep Mini/APP and AllPrep Micro/Acetone vs Urea) and sample input amounts (25 vs 250 µg) with technical replicates of each condition tested. The quality of each experimental setting was assessed based on the number of substrates detected. As shown in Supplementary Fig. 3, RNALater and snap frozen provided a similar yield of substrates. However, sample preparations with urea gave better results than commercial protein isolation kits. As it was expected, the higher concentration of protein input was related to the higher number of substrates detected. These results indicate that the amount of input protein could be as low as 25 µg while urea-based protein extraction is used.

### Proteome and Phosphoproteome sample preparation and analysis

Proteomics and phosphoproteomics were performed using mass spectrometry at the mass spectrometry facility at Barts Cancer Institute as previously reported [3, 18] with minor modifications. Frozen tissue was homogenised in 8 M urea buffer supplemented with phosphatase inhibitors (10 mM Na_3_VO_4_, 100 mM β-glycerol phosphate and 25 mM Na_2_H_2_P_2_O_7_ (Sigma-Aldrich)). Protein concentration in the supernatants was calculated by Bradford analysis. 25 µg protein were reduced and alkylated with 4.1 mM DTT and 8.3 mM IAA at room temperature for 15 min each. Proteins were digested into peptides by trypsinisation performed using 10 TAME (*p*-toluene-sulfonyl-L-arginine methyl ester) units of immobilised 1-chloro-3-tosylamido-7-amino-2-heptanone-trypsin for 16 h at 37 °C, and digestion stopped by the addition of TFA at a final concentration of 1%. Phosphopeptides were enriched from total peptides by a highly selective TiO2 chromatography enrichment procedure based on TiO2 micro-columns and peptide loading in 2,5-dihydroxybenzoic acid (DHB). The dried phospho-peptides and peptides, dissolved in 0.1% TFA, were analysed by a nanoflow ultimate 3000 RSL nano instrument coupled on-line to a Q-Exactive plus mass spectrometer (Thermo Fisher Scientific). Gradient elution was performed from 3 to 35% in 0.1% formic acid in acetonitrile in 120 min at a flow rate 300nL/min. A second buffer, 0.1% formic acid in water, was used to balance the mobile phase (spray voltage 1.95 kV, capillary temperature 255 ºC). The Q-Exactive Plus system was operated in data dependent mode with one survey MS scan followed by 15 MS/MS scans. Full scans were acquired in the mass analyser at 375-1500 m/z with the resolution of 70,000, and the MS/MS scans obtained with a resolution of 17,500. MS raw data files, converted into Mascot Generic Format (Mascot Distiller version 2.5.1), were searched against the SwissProt database (released December 2015), restricted to human entries, using the Mascot search daemon (version 2.5.0). Parameters include allowing mass windows of 10 ppm and 25 mmu for the parent and fragment m/z values, respectively, and a maximum of 2 missed cleavages. Fixed modification of carbomidomethyl (C) were considered and variable modifications included in searches were oxidation of methionine, pyro-glu (N-term) and phosphorylation of serine, threonine and tyrosine. Peptides with an expectation value < 0.05 were considered for further analysis. For peptide quantification, Pescal Software was used to construct extracted ion chromatograms for all the identified peptides across all conditions [3, 4]. Proteins were quantified using an in-house developed script which sums the intensities of all peptides comprised in the same protein and the data was normalised by the sum of all intensities from a sample. Only proteins with at least 2 different peptides and mascot score > 50 were considered for further analysis. Maximum intensity across technical replicates was considered for further analysis. Intensity values of zero (not detected) were substituted by minimum intensity values across all proteins in the same sample divided by 10. The data was further normalised by log_2_ transformation and quantile normalisation in R for downstream analysis. Raw mass spectrometry data was deposited in the ProteomeXchange Consortium via the PRIDE partner repository [19] with the dataset identifier: PXD044963. Sample details for both total proteome and phosphoproteome are given in Supplementary Table 2.

### Bioinformatic analysis

Differential protein and phosphosite levels were determined using limma R package (version 3.50.1) and *p*-values were corrected using the qvalue package (version 2.26.0) from Bioconductor. The ComplexHeatmap (version 2.12.1) Bioconductor package was used to perform Ward’s hierarchal clustering with Euclidean distance matrix on Z-score transformed data. Kinase substrate enrichment analysis was performed using the fry function from the limma package and a comprehensive resource of kinase to substrate mappings from 3 databases (PhosphoSitePlus [20], PhosphoNetworks [21], and KEA2 [22]). The mappings were downloaded from their online websites during June 2022 and reformatting was performed with Python. Pearson correlation between clinical endpoints and proteins/phosphosites were undertaken using the ‘cor.test’ function in the stats (version 4.2.0) R package. Like kinase analysis, functional enrichment was undertaken using ‘fry’ function and the proteins corresponding to the differentially expressed peptides from the proteome and phosphoproteome analysis separately. Pathway gene sets were downloaded from the KEGG database. Transcription factor (TF) activity was predicted using Virtual Inference of Protein-activity by Enriched Regulon analysis package (VIPER version 1.30.0) from Bioconductor. We included only TFs with confidence levels A, B and C as this combination has a reasonable balance between TF coverage and performance [23]. This subset of the TF-target interaction database contains 271 TFs and 5321 target genes. Since our proteomic data was covering a very small fraction of TF-target protein sets (2% of TFs and 7% of target proteins), for TF activity analysis, we considered genes as proxies of proteins and used gene expression data of the same samples that are publicly available [13]. The limma package was used to assess the statistical significance of TF activities between conditions compared. For variant calling from RNA-seq, we used standard GATK4 pipeline [24]. Prior to variant calling, sequencing reads were trimmed to remove the Illumina adapters using bbduk from the BBMap package v.38.94, with default parameters.

## RESULTS

### Phosphoproteomics reveals a clear distinction between the lymphoid and myeloid pathotypes

**Fig. 1:**
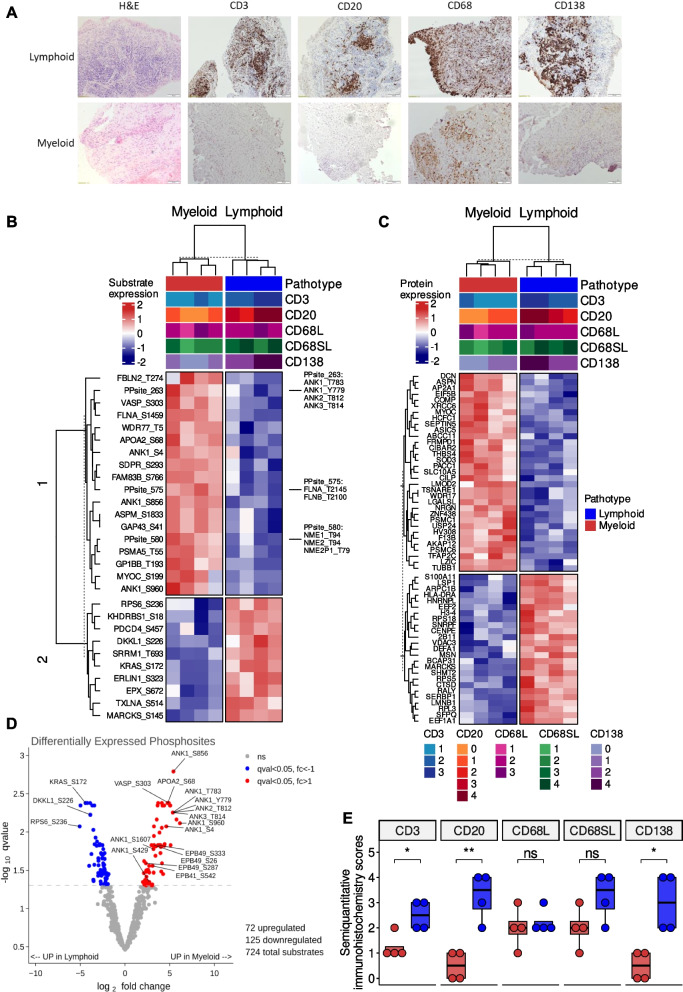
Clustering of differentially expressed phosphosites and proteins between the lymphoid and myeloid pathotypes. **A** Immunohistochemistry of synovial biopsies for CD3, CD20, CD68L, CD68SL and CD138 cell surface markers to classify samples as lymphoid (B cell aggregates present), myeloid (sublining macrophage infiltration). **B** Hierarchical clustering using the most significant phosphosites that were differentially expressed between the pathotypes (q < 0.01) and (**C**) the most significant proteins that were differentially expressed between the pathotypes (q < 0.05). Phosphosites with the similar levels were grouped for the visualisation of the heatmap. **D** Volcano plots of differentially expressed substrates between the pathotypes using limma comparing. **E** Boxplots showing semiquantitative histological scores of CD3, CD20, CD68L, CD68SL and CD138 for lymphoid and myeloid pathotypes. For statistical analysis, two-sided Wilcoxon signed-rank test was used. ns *p* > 0.05, * *p* < 0.05, ** *p* < 0.01

The phosphoproteome and total proteome profiles from 8 synovial biopsies, producing 25 µg protein, were characterised using label-free mass spectrometry. The demographics of the patients whom the synovial biopsies are derived from are described in Supplementary Table 1. There were no statistically significant differences in demographics or baseline disease characteristics between lymphoid and myeloid groups. However, we noted non-significant higher levels of ESR and ultrasound scores in lymphoid biopsy individuals consistent with previously published work [16]. The phosphoproteome analysis identified and quantified 724 phosphosites and the proteome analysis quantified 2,714 proteins. Of the 8 synovial biopsies, 4 were classified as lymphoid and the other 4 samples were myeloid based on semiquantitative scores (0–4) derived from immunohistochemical staining of CD3 (T cells), CD20 (B cells), CD68 (macrophages) and CD138 (plasma cell) cell surface markers (Fig. 1A).

Principal component analysis of both the phosphoproteome and proteome profiles demonstrated a clear separation between the two pathotypes: myeloid and lymphoid (Supplementary Fig. 1A-B). Differential analysis of the phosphosite and protein levels between the myeloid and lymphoid pathotypes identified 198 phosphosites and 94 proteins that were differentially expressed between the two pathotypes (q-value < 0.05, Supplementary Table 3–4). Cluster analysis was performed on the phosphoproteome and proteome profiles, filtered for the top differentially expressed phosphosites (q-value < 0.01) and proteins (q-value < 0.05), respectively, and two clear clusters according to pathotype were detected (Fig. 1B-C). Upper tracks of heatmaps show pathotype, and histological scores for CD3, CD20, CD68L, CD68SL and CD138. Observed clusters were in line with the immunohistochemistry based manual pathotype classifications, CD3 + (T cells), CD20 + (B-cell) and CD138 + (plasma cells) were significantly higher in lymphoid pathotype compared to myeloid (Fig. 1E).

For the phosphoproteome profile, specific phosphosites found upregulated in the lymphoid pathotype include Kirsten Rat Sarcoma (KRAS-Ser172), Ribosome Protein S6 (RPS6-Ser236), and Dickkopf Like Acrosomal Protein 1 (DKKL1-Ser226) whereas specific phosphosites found upregulated in the myeloid pathotype include Apolipoprotein A2 (APOA2-Ser68), Vasodilator Stimulated Phosphoprotein (VASP-Ser303), several phosphosites of Ankyrin (ANK) family proteins (ANK1-Ser1607/Ser4/Ser429/Ser781/Ser856/Ser960/Thr1380/Thr1684/Tyr779, ANK2-Thr812, and ANK3-Thr814) (Fig. 1D).

For the proteome profile, upregulated proteins in the lymphoid pathotype include Lymphocyte Specific Protein 1 (LSP1) and HLA Class II Histocompatibility Antigen, DR Alpha Chain (HLA-DRA), which are both known to be highly expressed in T and B cells [25]. Similarly, proteins with higher expression in the myeloid pathotype include thrombospondin-4 (THBS4), myocilin (MYOC) and decorin (DCN). Interestingly, THBS4 expression in macrophages has been associated with inflammation [26] and MYOC is a critical ligand that regulates various signalling pathways, including Wingless/Integrated (WNT) and RAS Homologous (RHO) [27, 28]. DCN is an extracellular matrix proteoglycan which is known to interact with receptor tyrosine kinases, such as Epithelial Growth Factor Receptor (EGFR), Vascular Growth Factor Receptor (VEGFR) and Hepatocyte Growth Factor Receptor (MET) [29].

We also compared the protein expression levels of the phosphosites differentially expressed between the pathotypes to show that the phosphosite measurements were not biased towards protein expression levels. We could perform this comparison only for the proteins present in both datasets. Supplementary Fig. 2 shows the statistical significance and fold change levels of proteins corresponding to 13 out of 28 significant phosphosites given in the Fig. 1B.

### Receptors, ligands and kinases are associated with pathotypes

**Fig. 2:**
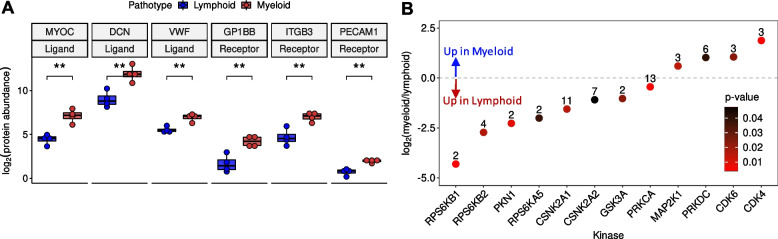
Receptor and ligand expression levels and predicted kinase activity between lymphoid and myeloid pathotypes. **A** Boxplots of the differentially expressed ligands myocilin; (MYOC), decorin (DCN) and Von Willebrand Factor (VWF) and the differentially expressed receptors; Platelet Endothelial Cell Adhesion Molecule (PECAM1), Glycoprotein Ib Platelet Subunit Beta (GP1BB) and Integrin Subunit Beta 3 (ITGB3). **B** Scatterplot illustrating the predicted differential kinase activities between lymphoid and myeloid pathotypes

To gain a deeper understanding of the potential signalling drivers associated with the pathotypes, the significant differentially expressed proteins were datamined for known receptors and ligands. As well as the previously mentioned MYOC and DCN, Von Willebrand Factor (VWF) was highlighted to be upregulated ligands in the myeloid pathotype compared to lymphoid (Fig. 2A). Furthermore, in the myeloid pathotype, there was the upregulation of the receptors: Platelet Endothelial Cell Adhesion Molecule (PECAM1), Glycoprotein Ib Platelet Subunit Beta (GP1BB) and Integrin Subunit Beta 3 (ITGB3) (Fig. 2A). Notably, PECAM1 is a receptor for VWF.

In addition, kinase substrate enrichment analysis was undertaken using the significant differential phosphosite levels between the two pathotypes (Fig. 2B, Supplementary Table 5). Since publicly available phosphoproteomic databases of kinase-substrate mappings are incomplete, we reformatted and merged three main human databases (PhosphoSitePlus [20], PhosphoNetworks [21] and KEA2 [22]) to obtain a more comprehensive database. This new database has a total of 28,319 (16,464 unique) substrates that map to 457 kinases (Table 1). From this analysis, kinases related to the Mammalian Target Of Rapamycin (MTOR) pathway were predicted to be highly active in the lymphoid pathotype, such as Ribosomal Protein S6K (RPS6K) family members (RPS6KB1/B2/A5). Lymphoid enriched phosphosites associated with RPS6K, which include RPS6-Ser236, Programmed Cell Death 4 (PDCD4-Ser457) and Heterogenous Nuclear Ribonucleoprotein A1 (HNRNPA1-Ser4/Ser6) [30].

**Table 1:** Combining public kinase-substrate human databases. Breakdown of kinase-substrate pairs collected from PhosphoSitePlus, PhosphoNetworks and KEA2 to construct a more comprehensive database

Database	Number of kinases	Number of substrates	Multi-mapping substrates	Unique substrates	Accessed/Created on
**KEA2**	250	13886	6030	7856	Jun-22
**Phosphonetwork**	230	4417	1826	2591	Jun-22
**Phosphositeplus**	407	14274	4623	9651	Jun-22
**Combined**	457	28319	11855	16464	Jun-22

Also, Serine/Threonine Protein Kinase N1 (PKN1), Casein Kinase II Subunit α 1/2 (CSNK2A1/2), Glycogen Synthase Kinase 3 α (GSK3A) and Protein Kinase C α (PRKCA) were predicted to be highly active in the lymphoid pathotype based on assessment of their substrate enrichments, but these kinases have not been relatively well-explored in the context of RA disease or as a therapeutic target.

On the other hand, Cyclin Dependent Kinase 4/6 (CDK4/6) were predicted to be upregulated in the myeloid pathotype along with Protein Kinase DNA-Activated, Catalytic Subunit (PRKDC) and Mitogen-Activated Protein Kinase Kinase 1 (MAP2K1). Filamin A (FLNA-Ser1459) and WD Repeat Domain 77 (WDR77-Thr5) are myeloid enriched substrates associated with CDK4/6 and potential upregulation of CDK4/6 may be a downstream effect of the predicted increase in MAP2K1 activity [11].

### Immunological pathways are preferentially associated with the lymphoid pathotypes

**Fig. 3:**
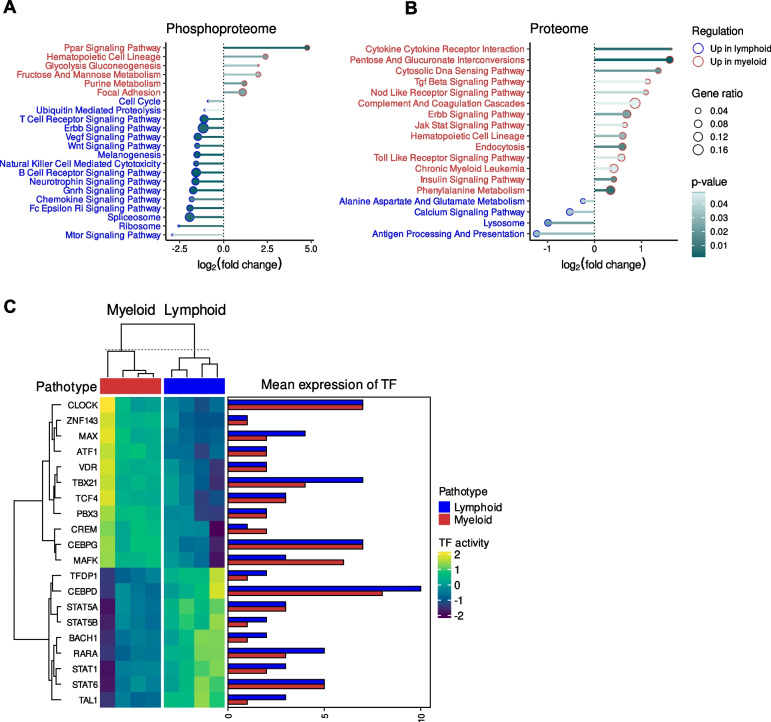
Pathway and transcription factor enrichment analysis of the differential phosphosite and protein levels between the lymphoid and myeloid pathotype. **A** Dotplot of the Kyoto Encyclopedia of Genes and Genomes (KEGG) pathway enrichment analysis using the differential phosphosite levels between the two pathotypes and (**B**) the differentially expressed proteins between the two pathotypes. **C** Heatmap of the predicted transcription factor activity based of the differentially expressed proteins between the pathotypes and a barplot of the mean transcription factor expression for each pathotype

Pathway enrichment analysis was undertaken on the significant protein expression differences between the two pathotype proteome profile and the proteins associated with the significant differential phosphosite levels between the two pathotype phosphoproteome profiles (Fig. 3A-B). Furthermore, transcription factor activities were inferred from the matched RNA-seq gene expression profiles of samples (Fig. 3C).

The phosphosites upregulated in the lymphoid pathotype were enriched with the MTOR signalling pathway term (Fig. 3A), which corroborates the predicted upregulation in the activities of RPS6K family members in the lymphoid pathotype. There were also enriched terms relating to immune signalling and activity at both the phosphoproteome and proteome level, which corresponds to the lymphoid pathotype being the most immunologically active as previously shown in RNA-seq analyses [13]. For instance, there was enrichment in the lymphoid phosphoproteome of T cell receptor signalling pathway, B cell receptor signalling pathway, natural killer cell mediated cytotoxicity and Fc Epsilon Ri signalling pathway (Fig. 3A). Similarly, there was the enrichment of the antigen processing and presentation pathway for the lymphoid proteome profile (Fig. 3B). However, it was noted that the annotation of JAK-STAT signalling pathway was more enriched in the myeloid proteome profile. In comparison, transcription factor activity analysis of RNA-seq data highlighted the potential upregulation of key transcription regulators of immune and inflammatory response (Fig. 3C), such as CCAAT/Enhancer-Binding Protein δ (CEBPD) and STAT family members (STAT1/5A/5B/6) in the lymphoid pathotype.

In contrast, the myeloid pathotype was enriched with the focal adhesion term in the phosphoproteome profile (Fig. 3A), which coincided with the upregulation of ITGB3 expression. The myeloid pathotype was also enriched with annotations relating to platelets, which corresponds with the myeloid lineage and higher expression of PECAM1 in the myeloid group. For example, there was the upregulation of the PPAR signalling pathway in the phosphoproteome profile (Fig. 3A) whereas there was the enrichment of complement and coagulation cascades annotation in the proteome profile (Fig. 3B). Notably, there were other signalling pathway annotations that were highlighted in the enrichment analysis of the myeloid proteome profile (Fig. 3B), which include Transforming Growth Factor β (TGFβ) signalling, Nucleotide Binding Oligomerization Domain (NOD) like receptor signalling pathway, Erythroblastic Oncogene B (ERBB) signalling pathway, JAK-STAT signalling pathway, toll like receptor signalling pathway and insulin signalling pathway.

Transcription factor activity analysis of the myeloid pathotype (Fig. 3C) highlighted MYC associated factor X (MAX), which perhaps corresponds to the enriched MAP2K1 and CDK4/6 kinase activity because downstream of MAPK/ERK signalling, MAX dimerises with MYC and both transcription factors regulate CDK [31]. The myeloid pathotype also had potential upregulation of key transcriptional regulators of immune cells and cytokine expression. For instance, there was the enrichment of T-Box Transcription Factor 21 (TBX21) and CCAAT/Enhancer Binding Protein γ (CEBPG). TBX21 regulates helper T cell differentiation [32] whilst CEBPG facilitates interleukin-4 expression [33].

### Correlation between clinical endpoints and phosphosites

**Fig. 4:**
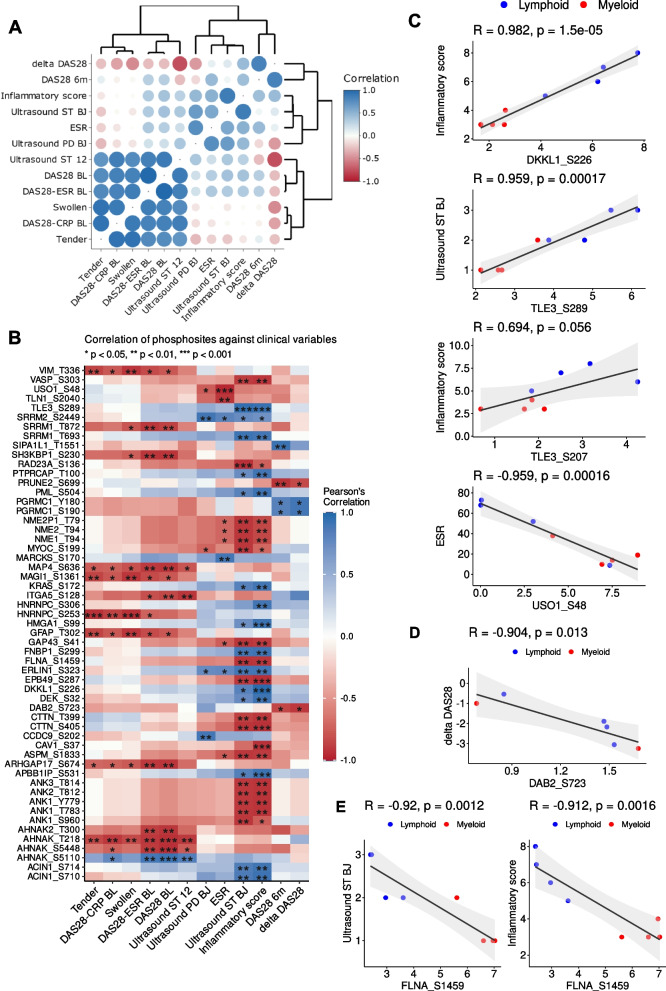
Correlation between clinical endpoints and phosphosite levels. **A** Correlation-based similarities of clinical variables. **B** Heatmap of the Pearson correlations between phosphosite levels and clinical endpoints (ESR: erythrocyte sedimentation rate; CRP: C-reactive protein; Ultrasound ST/PD BJ: ultrasonographic scores (ST, synovial thickness; PD, power doppler) at the biopsy joint (BJ); Ultrasound ST/PD 12: ultrasonographic scores across 12 representative joints, disease activity scores: DAS28-CRP/ESR baseline and DAS28 (baseline, after 6 months and the delta between baseline and 6 months). Correlation pairs with *p* < 0.05 and an absolute correlation coefficient higher than 0.9 are shown. Clinical endpoints at the x-axis are grouped and ordered based on similarities among them. **C** Pairs of clinical endpoints and phosphosites with the highest positive and negative correlation coefficients. **D** Inverse correlation between DAB2-Ser723 with delta DAS28. Two missing delta DAS28 data points were not shown in this plot. **E** Correlation of FLNA-Ser1459 with ultrasound score synovial thickening and inflammatory score

Pearson correlation was undertaken between various clinical endpoints and the phosphosites from the phosphoproteome profiles, respectively, to investigate potential biomarkers of disease activity or response. Clinical endpoints investigated in the correlation analysis include: number of tender/swollen joints, erythrocyte sedimentation rate (ESR), Krenn inflammatory score, ultrasound power Doppler (PD) and synovial thickness (ST) at the biopsy joint, Disease Activity Scores (DAS)-28 including DAS28-CRP/ESR at baseline and delta DAS28 (the delta between baseline and 6 months DAS28-ESR score). It was noted that there are high positive correlations between ultrasound ST, number of tender/swollen joints and baseline DAS28, DAS28-CRP/ESR (Fig. 4A).

Several phosphosites had significant correlation to the clinical endpoints (Fig. 4B) and some of these were also associated with specific pathotypes where 25% of the differentially expressed substrates were well correlated (*p* < 0.05 & |r|> 0.9) with at least one clinical endpoint mentioned above. For instance, Krenn inflammatory score against DKKL1-Ser226, ultrasound score for synovial thickness at the biopsy joint and inflammatory score against Transducing-Like Enhancer Protein 3 (TLE3)-Ser289 showed the highest positive correlations, and ESR against General Vesicular Transport Factor P115 (USO1)-Ser48 had the highest inverse correlation (Fig. 4C).

The majority of the phosphosites identified do not have a known associated kinase, including DKKL1-Ser226, TLE3-Ser289 and USO1-Ser48. Nonetheless, it was noted that FLNA-Ser1459 is a target of CDK1/4 that is known to facilitate cell motility and cytoskeletal remodelling [34] and FLNA-Ser1459 was found in our analysis to be negatively correlated with ultrasound score synovial thickening and inflammatory score (Fig. 4E). Higher levels of FLNA-Ser1459 were associated with myeloid and CDK4/6 was predicted to be enriched in the myeloid pathotype (Fig. 2B).

Interestingly, there was also a negative correlation between Disabled Homolog 2 (DAB2)-Ser723 with delta DAS28 (Fig. 4D). DAB2-Ser723 is a downstream target of the kinases ROCK2 and CAMK2G and this specific phosphosite has been associated with integrin activation in thrombin signalling in human platelets [35].

### Identification of potential missense variants underlying RA pathotypes

RNA-seq is a high-throughput method that is primarily designed for the quantification of mRNA gene expression using sequencing reads. Because it is based on a deep sequencing technique and requires mapping of reads against to reference genome, it enables observation of single nucleotide variations (SNV) that produce amino acids that are different from the usual amino acids at those positions. With the combination of our PEAC transcriptomics study [13] and our proteomic analysis, we applied the variant calling pipeline using GATK4 and analysed the missense variants that can alter the functions of proteins, which may affect the RA pathways and pathogenesis. The association of the pathotype with the genotype was examined using the chi-squared test. The allelic frequency of rs247860 (c.*104A > G) that is in the 3'UTR region of Coactosin-Like F-Actin Binding Protein 1 (COLT1) was significantly higher in lymphoid samples compared to myeloid (*p* = 0.034). COLT1 gene encodes one of the numerous actin-binding proteins which regulate the actin cytoskeleton, and it is known to be related to innate immune system pathways [36]. In myeloid samples, three SNVs and one deletion showed higher proportions compared to lymphoid samples (Supplementary Table 6). For instance, rs1051420 (c.*651A > C) in the 3'UTR region of ETS Proto-Oncogene 2 (ETS2) was frequently acquired in myeloid samples. ETS2 is a ubiquitous transcription factor activated after phosphorylation at threonine-72 residue [37] and its active form induces the molecular reprogramming of rheumatoid arthritis synovial fibroblasts (RASFs) to osteoclast-like cells that contribute to cell population heterogeneity in the synovium [38]. However, since the population-specific frequencies reported in gnomAD Database (Supplementary Table 6) are higher than 0.1 and there is no clinical significance reported yet in the ClinVar database, it is difficult to relate this SNV directly with RA diverse clinical phenotypes. Missense mutations can affect transcription factors resulting in altering the expression of the corresponding protein. Supplementary Fig. 4 shows the negative correlation (*r* = -0.6) between protein abundance and mRNA expression level of Glycoprotein Ib Platelet Subunit Beta (GP1BB). The SNV in this gene, rs1059196 (c.*107C > T), may be associated with its expression levels in synovial tissue which explains the inverse correlation between gene and protein expression levels.

## Discussion

In this study, we conducted a pilot phosphoproteome and total proteome profiling of early RA synovium to elucidate how the signalling networks differ between different RA histopathological phenotypes and their correlation with clinical and imaging parameters. Although RNA-seq has significantly advanced our understanding of RA tissue biology and pathogenesis [13, 17, 39], it can only measure mRNA levels and not protein levels. For a small number of proteins, mRNA levels are well known to display poor correlation to the amount of that protein in the tissue – cytokines are a notorious example of this and often show very low mRNA levels since they are stored in granules and then released, and the transcript only reflects resynthesis. Mass spectrometry-based proteomics has the advantage of measuring protein levels directly. Furthermore, phosphoproteomics has the potential to distinguish activation states of proteins and thus quantify whether key signalling proteins are phosphorylated and in an active versus inactive state. In oncology, phosphoproteomics has been able to elucidate signalling pathways which determined sensitivity or resistance to kinase inhibitors [3, 4].

It was clear from the differential analysis of the phosphoproteome and total proteome profiles that the synovium has distinct active signalling signatures according to the lymphoid or myeloid prevalent pathotype, which corroborates our observations with the RNA-seq analysis of early RA synovium [13]. Similar to the RNA-seq analysis, differentially expressed proteins and pathway enrichment analysis demonstrated that the lymphoid pathotype was more immunologically active compared to the myeloid pathotype due to the upregulation of proteins associated with lymphocytes, such as LSP1, and the upregulation of cellular mechanisms relating to immune signalling and activities at both the phosphoproteome and proteome level, such as Fc Epsilon Ri signalling pathway and antigen processing and presentation.

Complementing the previous RNA-seq analysis of PEAC samples [13], kinase enrichment analysis of the differentially expressed phosphosites offers deeper insights into the potential signalling networks associated with the lymphoid and myeloid pathotypes, which could be exploited by kinase targeted therapeutics. For example, the lymphoid pathotype had an enrichment of substrates for kinases involved in the MTOR signalling pathway (RPS6K family members). This cellular mechanism is known to be critical in B cell activation and development [30], so its increased activity in the lymphoid pathotype suggests its potential fundamental role in maintaining a more severely inflamed state within RA synovium. Currently, mTOR inhibitors are clinically approved for various cancer types, such as everolimus [40], but it is not approved in RA patients. A clinical trial has demonstrated the beneficial combination of everolimus and methotrexate in RA patients compared to methotrexate alone [41]. However, the combination did increase ESR despite the decrease in swollen/tender joints and this pro-inflammatory side effect from mTOR inhibition may be due to the complexity of the signalling pathway, particularly with the multiple interactions with various cell types and the different cell populations in the synovium between patients. Further investigations into the importance of MTOR signalling in the myeloid pathotype are required to determine its potential as a target for patients with this pathotype compared to the lymphoid rich patients.

Conversely, CDK inhibition could represent a useful therapeutic strategy for RA patients with the myeloid pathotype who are enriched for this specific kinase. This could be a downstream effect of the ITGB3 and MAP2K1, which were also highlighted to be upregulated in the myeloid pathotype. However, specific probing of particular phosphosites involved in these pathways, especially those on the lowly abundant tyrosine residues, would be required to determine its real potential as a therapeutic target. Interestingly, high levels of FLNA-Ser1459, a CDK4/6 target, is negatively correlated with ultrasound score synovial thickening and so could represent a potential biomarker of RA with a myeloid pathotype with a lower inflammatory state. Currently, CDK4/6 inhibitors have been approved for breast cancer [42] and has been shown to suppress RA in animal models [11]. Although CDK inhibitors are not approved for RA and are currently under pre-clinical investigation, there is potential that CDK4/6 inhibitors may be more relevant to RA individuals with the myeloid pathotype given its enrichment in this study. Furthermore, as mentioned, the results of an early phase trial of Seliciclib, an orally available CDK inhibitor in patients with active rheumatoid arthritis, has been reported [12]. CDK2 and CDK6 have also been reported as risk loci associated with RA [43] which highlights their influence in RA.

Notably, in addition to identifying known kinase activities associated with RA that could be potentially targeted to specific RA pathotype, our phosphoproteome analysis revealed potential RA-associated kinases that are understudied, including PKN1 increased in the lymphoid pathotype and PRKDC in the myeloid pathotype. PKN1 is a fatty acid activated serine/threonine kinase and has been detected to interact with TNF Receptor-Associated Factor 2 (TRAF2) in a yeast two-hybrid study [44]. TRAF2 modulates Tumour Necrosis Factor Receptor Superfamily (TNFRSF) mediated activation of NF-kB, which suggests that PKN1 has a role in NF-kB mediated inflammation. PRKDC is a critical DNA-dependent serine/threonine kinase which regulates the DNA damage response, but it has also been demonstrated to regulate immune response through the cyclic GMP-AMP-Stimulator Of Interferon Genes (cGAS-STING) pathway and *PRKDC* mutations have been associated with autoimmune disease [45, 46]. To the best of our knowledge, the therapeutic potential of PKN1 and PRKDC has not been investigated in RA. However, given recent reports in the oncoimmunology field [47], they could be considered as novel therapeutic targets.

This pilot study detected several key kinases known to be important in RA which showed distinct phosphorylation activity between the myeloid and lymphoid pathotypes. However, the majority of phosphosites identified in the phosphoproteome analysis do not have a known associated kinase. Current databases of kinase-substrate mappings are heavily based on cancer studies [20–22] and although kinase-substrate mapping is likely to overlap with RA, it is plausible that many synovial tissue specific mappings remain to be discovered. For instance, KRAS-Ser172 was identified to upregulated in lymphoid pathotype and KRAS is a RAS family member, which is a key regulator of MAPK, AKT/MTOR and RAL Guanine Nucleotide Dissociation Stimulator (RAL-GDS) signalling [48] so there is the possibility that this phosphorylation modification could modulate pathotype specific signalling networks. In particularly, the regulation of KRAS may determine the potential upregulation of MTOR signalling in the lymphoid pathotype compared to the predicted upregulation of the MAP2K1 activity in the myeloid pathotype.

Besides characterising the signalling signatures associated with the lymphoid and myeloid pathotype, this pilot phosphoproteome study reveals the link between the phosphoproteome signatures and disease activity and response. Besides the aforementioned negative correlation between FLNA-Ser1459 and ultrasound score synovial thickening, DAB2-Ser723 showed negative correlation against disease activity score, delta DAS28. DAB2 regulates macrophage polarisation towards the M2 phenotype through inhibiting NF-κB signalling [49], and DAB2 expression was found to be exclusive to FOXP3 positive regulatory T cells amongst the T cell population and is thought to facilitate gap junctions with CD4 + effector T cells [50]. Although the role of DAB2 in immune response is in its infancy, it is possible that DAB2-Ser723 may have a multi-faceted function in regulating different immune cell populations in RA, influencing differences in disease activity.

For a more informative analysis on the dynamic nature of biomolecules and potential mechanisms behind diseases, proteomics can also be incorporated with transcriptomics and other -omic information. In this study, alongside the proteomics, we used pair-end RNA-seq data for gene expression quantification and also for variant calling. Our integration of RNA-seq and proteomics allowed us to speculate that SNVs may influence protein levels and function and so are contributing factors in affecting the signalling profiles of RA, thus affecting disease pathogenesis. For instance, rs1059196, a nucleotide substitution at the stop codon appears to affect alternative splicing of *SEPT5-GP1BB* readthrough, which also contributes to the explanation of the inverse correlation between protein and gene expression of this molecule. Furthermore, our approach also highlighted SNVs that were commonly acquired in certain pathotypes. In particular, two missense variants, rs247860 and rs1051420 that are in *COLT1* and *ETS2* genes respectively, were discovered to be pathotype-specific. Early reports suggest that polymorphisms of *COLT1* might be associated with the genetic susceptibility of autoimmune disorders such as RA and systemic lupus erythematosus (SLE) [36]. ETS has a critical role in molecular reprogramming of synovial fibroblasts (RASFs) to osteoclast-like cells that contribute to cell population heterogeneity in the synovium. However, given the high allele frequencies of these SNVs recorded in large-scale sequencing repositories, we could only hypothesize that both SNVs may affect cell differentiation, which connects these two SNVs to RA indirectly through the synovial tissue's cellular composition. Thus, multi-omics profiling, rather than individual “omics” of synovial biopsies may represent the best way forward towards personalised medicine in RA.

Limitations of this phosphoproteomic study are the small number of samples analysed, but this was necessary as this was a pilot study to determine the feasibility of using synovial biopsy material for mass spectrometry, while a limitation of mass spectrometry is that it typically requires much larger amounts of material than other high throughput omics methods, of the order of micrograms vs nanograms. We tested whether the amount of input material could be reduced from 250 µg down to 25 µg and still generate informative phosphoproteomic signatures. Our study shows that not only this is feasible but also provides validation of phosphoproteomic analysis on synovial biopsies with highly informative results.

## Conclusions

Overall, this pilot study, despite its low sample size and limited statistical power, illustrates significant differences in the phosphoproteome and proteome profiles between the myeloid and lymphoid pathotypes which are distinct from those signatures previously identified in mRNA studies. This provides strong evidence in support of future larger scale phosphoproteomic analysis of the RA synovium. A conceivable hypothesis would be that RA individuals which showed low levels of activation of relevant downstream signalling proteins in disease tissue would be less likely to respond to drugs which target proteins or cell surface receptors associated with targeting those pathways. Further understanding of the heterogeneity of the signalling signatures in the synovium could lead identification of novel RA drug targets and could help identify novel signatures for stratification of RA patients to different drugs as part of a personalised medicine approach to therapy.

### Supplementary Information

Supplementary Material 1.

Supplementary Material 2.

## Data Availability

Raw mass spectrometry data was deposited in the ProteomeXchange Consortium via the PRIDE partner repository with the dataset identifier: PXD044963.
