# Supplementary material for: Phosphoproteomic profiling of early rheumatoid arthritis synovium reveals active signalling pathways and differentiates inflammatory pathotypes
Source: Arthritis Res Ther. 2024 Jun 12;26:120. doi: 10.1186/s13075-024-03351-4 (PMC11167927; doi:10.1186/s13075-024-03351-4)

Supplementary Table 1: Baseline demographics of the patients. Data are frequency counts or median values with % of samples or range in brackets, respectively. ESR=erythrocyte sedimentation. CRP= C-reactive protein. DAS28=28 joint count disease activity score. P values in comparing lymphoid and myeloid samples are provided where t-test was undertaken for numerical parameters and fisher exact test was undertaken for categorical parameters.

		All (n=8)	Lymphoid (n=4)	Myeloid (n=4)	P value (Lymphoid vs Myeloid)
Sex	Female	5 (62.5%)	2 (50%)	3 (75%)	1
	Male	3 (37.5%)	2 (50%)	1 (25%)	
Age		41 (29-72)	56.5 (37-72)	32 (29-45)	0.064
Onset (months)		5.5 (3-7)	5.5 (3-6)	5 (3-7)	1.000
ESR (mm/h)		33.5 (9-73)	60 (9-73)	21.5 (10-38)	0.154
CRP (mg/L)		19 (5-76)	21 (5-76)	19 (12-54)	0.808
DAS28ESR Baseline		5.5 (3.67-7.03)	5.705 (5.17-7.03)	5.44 (3.67-5.9)	0.263
DAS28CRP Baseline		5.28 (3.53-6.55)	5.145 (4.74-6.55)	5.305 (3.53-6.3)	0.711
Ultrasound 12-max score (synovial thickening)		22 (4-36)	22.5 (15-36)	16 (4-24)	0.215
Ultrasound (Power Doppler)		3.5 (0-13)	7 (0-13)	1.5 (0-4)	0.160
Previous methotrexate use		8 (100%)	4 (100%)	4 (100%)	NA
Previous prednisolone use		4 (50%)	2 (5%)	2 (5%)	1

Supplementary figure 1: Clustering of the phosphoproteome and total proteome profiles from synovial biopsies. (A) Principal component analysis (PCA) of the phosphoproteome profiles and (B) total proteome profiles.

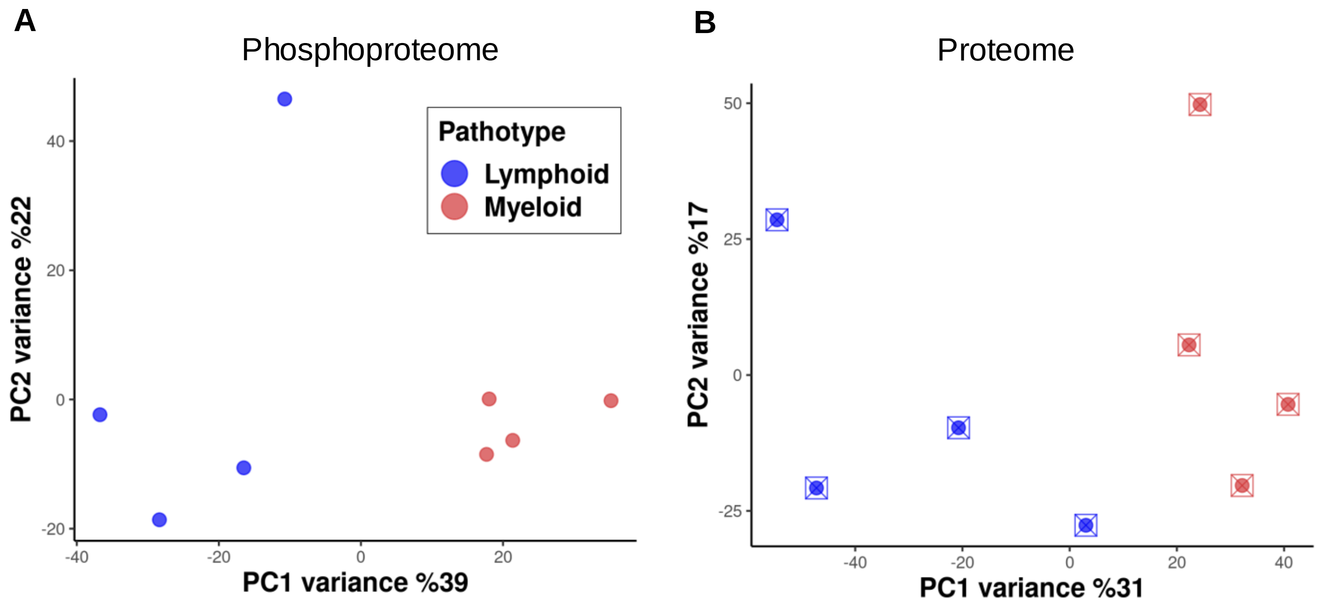

Supplementary figure 2: Independent expression patterns of proteins and phosphosites. Fold change levels of proteins that correspond to differentially expressed phosphosites shown in the Figure 2A.

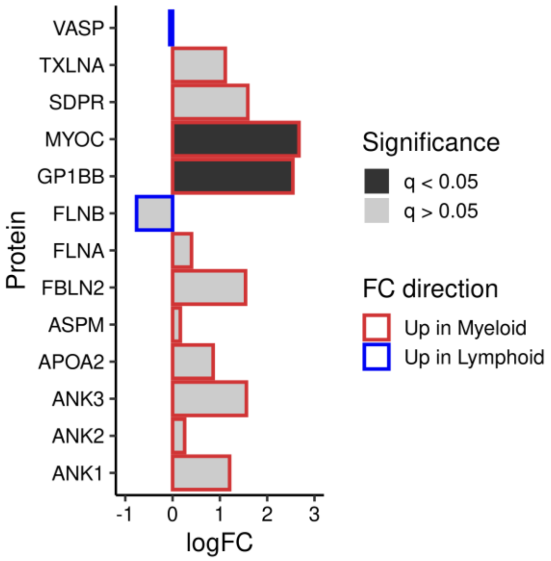

Supplementary figure 3: Results of the optimisation experiment. Boxplots showing median and first and third quartiles of the expression values of all phosphites quantified. Colours indicate different methods and concentrations benchmarked; AllPrep Mini/APP (blue), AllPrep Micro/Acetone (tan), Snap Frozen + Urea (red), RNALater + Urea (yellow), 25 µg (grey) and 250 µg (black).

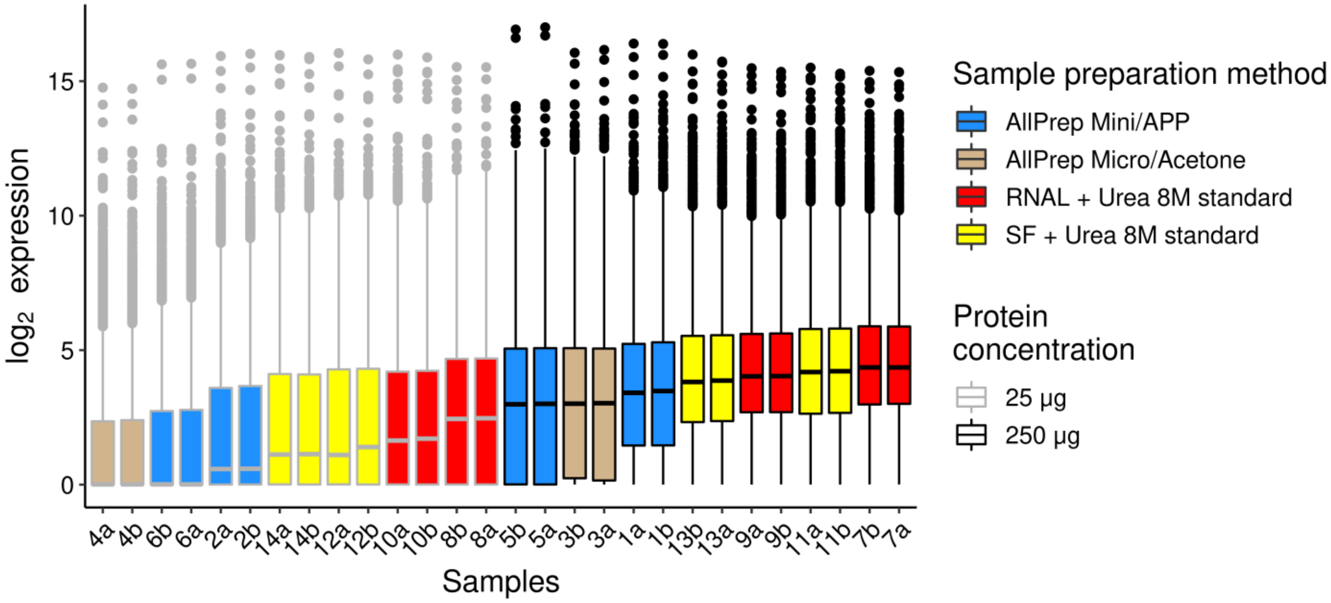

Supplementary figure 4: Protein and mRNA expression levels of GP1BB. Upper panel shows the concordance between protein and mRNA expressions of GP1BB, together for both Lymphoid and Myeloid (with higher allele frequency of rs1059196). Lower panel compares mRNA and protein expression levels between two pathotypes by two-sided t-test.

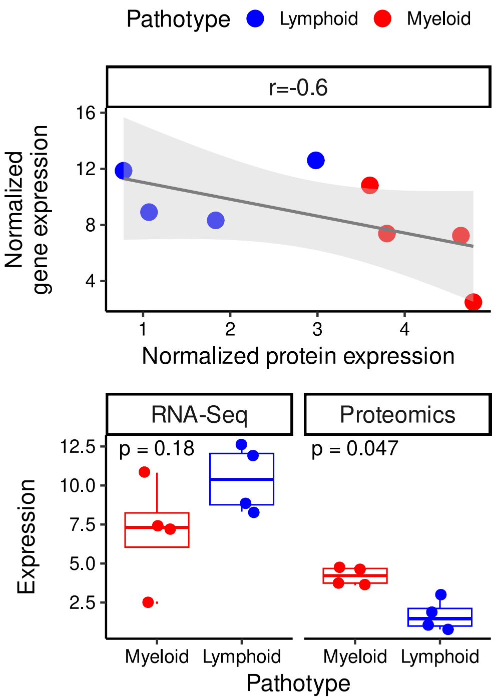

Supplement: Supplementary file 1 — Supplementary Material 1. [file 13075_2024_3351_MOESM1_ESM.pdf]
